# Fully semantic segmentation for rectal cancer based on post-nCRT MRl modality and deep learning framework

**DOI:** 10.1186/s12885-024-11997-1

**Published:** 2024-03-07

**Authors:** Shaojun Xia, Qingyang Li, Hai-Tao Zhu, Xiao-Yan Zhang, Yan-Jie Shi, Ding Yang, Jiaqi Wu, Zhen Guan, Qiaoyuan Lu, Xiao-Ting Li, Ying-Shi Sun

**Affiliations:** 1 Institute of Medical Technology, Peking University Health Science Center, Haidian District, No. 38 Xueyuan Road, Beijing, 100191 China; 2 Key Laboratory of Carcinogenesis and Translational Research (Ministry of Education/ Beijing), Department of Radiology, Peking University Cancer Hospital & Institute, Hai Dian District, No. 52 Fu Cheng Road, Beijing, 100142 China

**Keywords:** Deep learning, Different tumor regression grades, Post-nCRT MRI, Rectal cancer, Semantic segmentation

## Abstract

**Purpose:**

Rectal tumor segmentation on post neoadjuvant chemoradiotherapy (nCRT) magnetic resonance imaging (MRI) has great significance for tumor measurement, radiomics analysis, treatment planning, and operative strategy. In this study, we developed and evaluated segmentation potential exclusively on post-chemoradiation T2-weighted MRI using convolutional neural networks, with the aim of reducing the detection workload for radiologists and clinicians.

**Methods:**

A total of 372 consecutive patients with LARC were retrospectively enrolled from October 2015 to December 2017. The standard-of-care neoadjuvant process included 22-fraction intensity-modulated radiation therapy and oral capecitabine. Further, 243 patients (3061 slices) were grouped into training and validation datasets with a random 80:20 split, and 41 patients (408 slices) were used as the test dataset. A symmetric eight-layer deep network was developed using the nnU-Net Framework, which outputs the segmentation result with the same size. The trained deep learning (DL) network was examined using fivefold cross-validation and tumor lesions with different TRGs.

**Results:**

At the stage of testing, the Dice similarity coefficient (DSC), 95% Hausdorff distance (HD95), and mean surface distance (MSD) were applied to quantitatively evaluate the performance of generalization. Considering the test dataset (41 patients, 408 slices), the average DSC, HD95, and MSD were 0.700 (95% CI: 0.680–0.720), 17.73 mm (95% CI: 16.08–19.39), and 3.11 mm (95% CI: 2.67–3.56), respectively. Eighty-two percent of the MSD values were less than 5 mm, and fifty-five percent were less than 2 mm (median 1.62 mm, minimum 0.07 mm).

**Conclusions:**

The experimental results indicated that the constructed pipeline could achieve relatively high accuracy. Future work will focus on assessing the performances with multicentre external validation.

## Introduction

Colorectal cancer is the fourth most common cancer worldwide, with an annual incidence of more than 700,000 cases and the third-highest mortality rate [1]. According to the main international clinical guidelines [2, 3], the recommended treatment for locally advanced rectal cancer (LARC) is neoadjuvant chemoradiotherapy (nCRT), followed by total mesorectal excision (TME). In recent years, the watch-and-wait strategy appears to be a safer option in patients who have achieved pathologic complete response (pCR) after nCRT [4], while local excision, including transanal excision, transanal endoscopic microsurgery, and transanal minimally invasive surgery may be suitable for good response [5, 6]. At the same time, patients may also have the possibility of liver and pulmonary metastases [7, 8]. Therefore, accurate response prediction is essential in planning optimal treatment strategies [9–11].

As recommended by the guidelines, response assessment should be performed with the combination of restaging magnetic resonance imaging (MRI), digital rectal examination, and endoscopy, in which MRI plays an important role [12, 13]. However, the basic step for prediction is to accurately identify the residual tumor region or the tumor bed [14]. In general, the procedure is delineated manually by the radiologists on medical software, which is labor intensive and time-consuming [15]. As the essential modality of rectal cancer, T2-weighted imaging (T2WI) can display anatomical information with a clearer tumor boundary by high spatial resolution [16, 17]. Theoretically, patients accept MRI scanning before and after therapy to obtain baseline MRI (pre-nCRT MRI) and post-nCRT MRI [18]. Although pre-nCRT MRI is an important reference, its availability and accessibility is limited in real clinical practice. When conducting detection tasks only based on post-nCRT MRI images, the nCRT-induced submucosal edema, fibrosis, and/or mucin production make it difficult to distinguish changes after treatment from the residual tumor [19]. Meanwhile, the pathological changes induced by nCRT make the tumor appearance different from the primary counterpart in different tumor regression grades (TRGs) [20].

Some unsatisfactory and inaccurate results for restaging using standard manual MRI protocols [21] led to the need for a separate evaluation system for post-nCRT imaging. Currently, only a few of studies have used post-nCRT MRI for segmentation and prediction [22–24], but most are not based on the direct segmentation of lesions. The semantic segmentation for rectal cancer using the nnUNet framework [25–27] and post-nCRT single MRI modality has never been reported. The most commonly used medical image modality in former research is colon images scanned by computed tomography (CT) [28–30].

In this study, we explored and examined the segmentation potential for LARC exclusively on post-chemoradiation T2-weighted MRI using state-of-the-art deep learning (DL) architectures, with the aim to provide clinical auto-delineation tools for subsequent measurement and analysis [31–33]. Meanwhile, the generalization performance was further validated on tumor lesions with different TRGs. The quantitative metrics [34, 35], including Dice similarity coefficient (DSC), 95% Hausdorff distance (HD95), and mean surface distance (MSD), confirmed the practical implications of reducing workload whether for colorectal cancer physicians or radiologists.

## Methods

### Patients and dataset

**Fig. 1:**
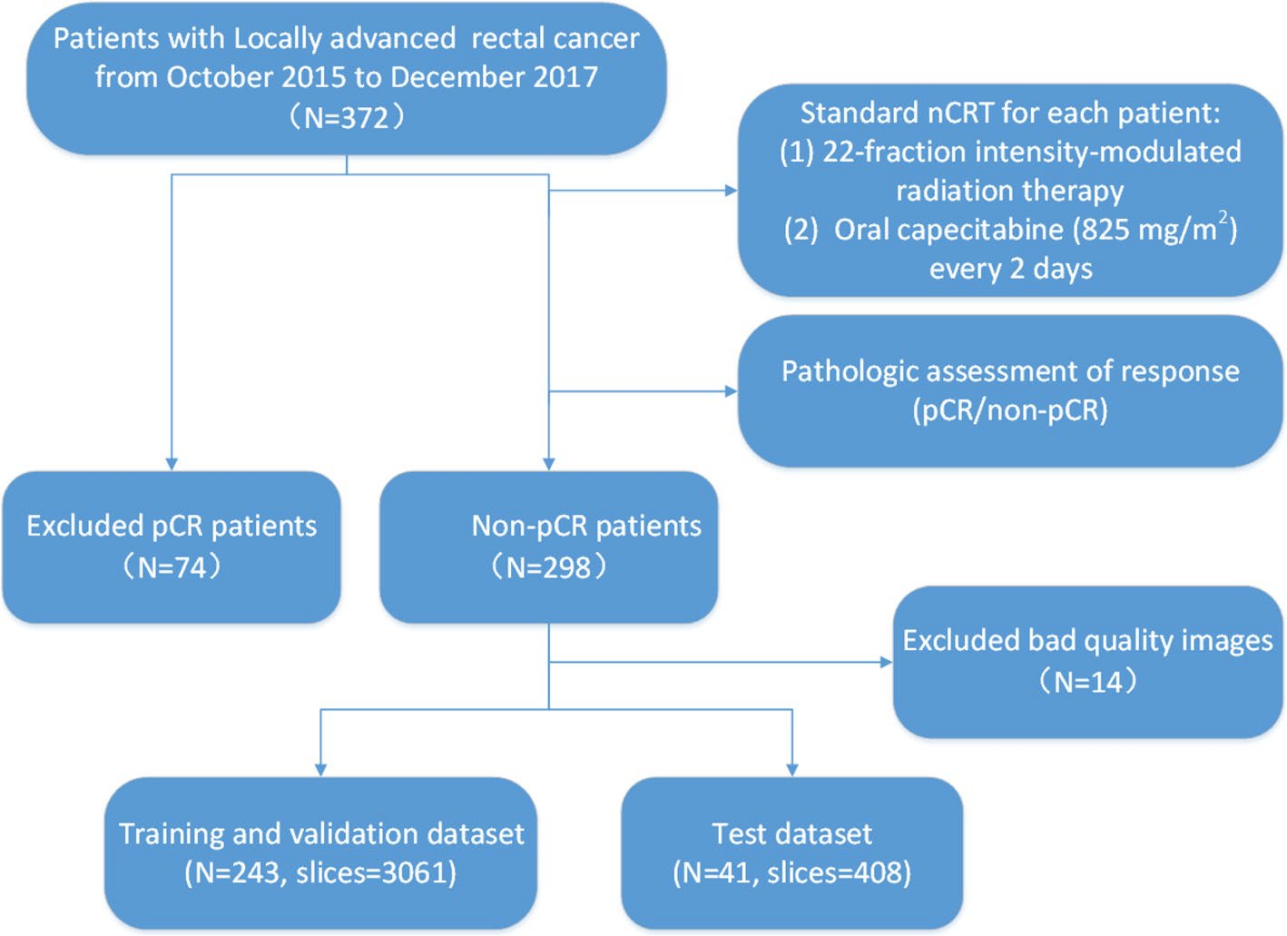
Flowchart showing the inclusion criteria for patients and the process of the overall dataset

The retrospective study enrolled 372 consecutive patients with LARC from October 2015 to December 2017. The inclusion criteria were as follows: (1) All candidates were pathologically confirmed with locally advanced rectal adenocarcinoma (excluding mucinous adenocarcinoma). (2) All candidates received a complete and standard nCRT process, which included 22-fraction intensity-modulated radiation therapy and oral capecitabine of 825 mg/m^2^ twice per day. (3) All candidates were scanned by T2-weighted MRI within 1 week before nCRT. (4) All candidates were scanned by T2-weighted MRI within 1 week before TME surgery. (5) All candidates were clinically confirmed to be in T3, T4, or N+ stage using baseline MRI. The clinical protocol was approved by the medical ethics committee of Beijing Cancer Hospital. Executing the process shown in Fig. 1, the overall dataset was produced containing rectal cancer images from 284 patients. Then, it was artificially grouped into training and validation dataset (*N* = 243), as well as test dataset (*N* = 41).

### MRI scan, image acquisition, and data preprocessing

All the post-nCRT MRI images were obtained with a 3.0-T MRI scanner (Discovery MR750; GE Healthcare, WI, USA). To minimize colonic motility for each patient, 20 mg of scopolamine butylbromide was administered intramuscularly 30 minutes before the MRI scan. A conventional rectal MRI protocol was applied to all patients, the standard process mainly included high-resolution T2WI from axial, coronal, and sagittal position, with diffusion-weighted imaging (DWI) as an auxiliary reference for subsequent delineation. And the main scan parameters are as follows: (1) High-resolution T2WI sequence: Repetition time (TR) =5,694 ms, repetition time (TE) =110 ms, field of view (FOV) = 180 × 180 mm, echo train length = 24, matrix = 288×256, thickness = 3.0 mm, and gap= 0.3 mm. (2) DWI sequence: Single-shot echo-planar imaging with 2 b-factors (0 and 1,000 s/mm^2^), TR = 2,800 ms, TE = 70 ms, FOV = 340 × 340 mm, matrix =256 × 256, thickness = 4.0 mm, and gap = 1.0 mm.

For the preprocessing steps, each volume was initially resampled to a consistent spatial resolution of 0.3516 × 0.3516 × 3.3 mm^3^ to ensure a uniform physical distance interpretation across acquired 3D images. The layers of each patient ranged from 18 to 40, with the same image size of 512 × 512 pixels. Then a total of 284 3D images were converted into 2D images using the SimpleITK package, and 3469 slices containing tumor lesions were screened to create the whole dataset. Each finished slice was stored in the NIfTI format (ie .nii.gz extension). To attain a standard normal distribution of image intensities, z-scores were utilized for the normalization (μ ± σ) of all the generated slices. At the final stage, 3061 slices were split to train the model with a random 20% internal validation set. Further, 408 slices were not involved in the model-building process for independent external validation.

### ROI delineation and manual annotation

The regions of interest (ROIs) on post-nCRT T2-weighted images were independently delineated by two experienced radiologists with 8 and 10 years of experience in abdominal radiology. And the ROIs were defined as all the residual tumors and suspected fibrotic areas. The lesion area on each slice was drawn along the tumor contour using ITK-SNAP v3.8.0 software. All the controversial images were reviewed by a third radiologist, and an agreement was reached if inconsistency existed in the judgment of tumor boundary details. The ROIs were created manually on T2-weighted images, the readers also referred to DWI images to avoid false positives or false negatives in the highest degree.

**Fig. 2:**
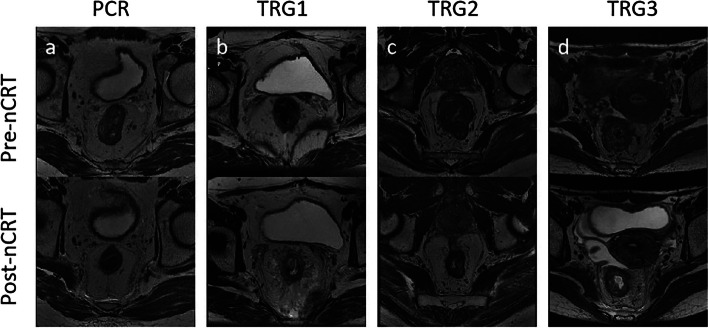
T2-weighted MRI images on pre-nCRT and post-nCRT. **a** pCR. **b** TRG1. **c** TRG2. **d** TRG3

After complete nCRT treatment and TME, surgically resected specimens were evaluated by two experienced pathologists with 10 and 15 years of experience in gastrointestinal disease, respectively. The annotations of TRG were referenced to the National Comprehensive Cancer Network and American Joint Committee on Cancer TRG system [36]. As shown in Fig. 2, the TRG indicator was defined into four levels (TRG0, TRG1, TRG2, and TRG3), and patients on TRG1, TRG2, and TRG3 were considered during model training and testing.

### Model construction: nnUNet framework for rectal tumor segmentation

**Fig. 3:**
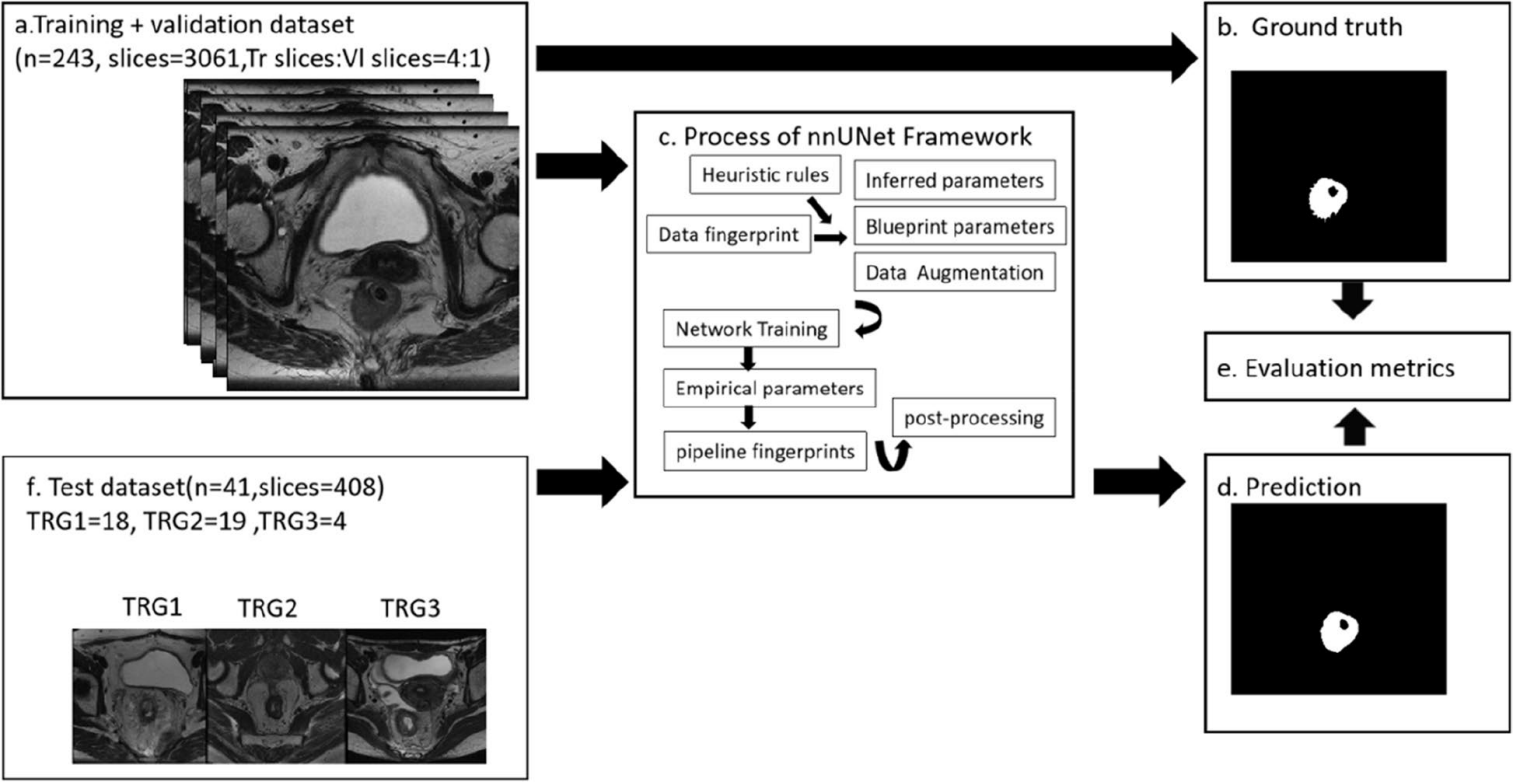
Overview of the deep learning modeling and evaluation flow. **a** Training and validation dataset (*n* = 243, slices = 3061, training slices:validation slices = 4:1). **b** Ground truth delineated by two radiologists. **c** Process of nnUNet Framework. **d** Prediction results using the trained DL model. **e** Evaluation metrics. **f** Test dataset (*n* = 41, slices = 408), TRG1 = 18, TRG2 = 19, and TRG3 = 4

nnUNet (https://github.com/MIC-DKFZ/nnUNet) is a general adaptive segmentation framework proven to have strong performance on 10 public datasets in international biomedical segmentation competitions (Liver Tumor, Brain Tumor, Hippocampus, Lung Tumor, Prostate, Cardiac, Pancreas Tumor, Colon Cancer, Hepatic Vessels, and Spleen) [25]. Merely regarding colorectal cancer segmentation, 190 CT images of colon cancer [37] were used in Medical Segmentation Decathlon (Memorial Sloan Kettering Cancer Center). However, the framework has not been widely applied to MRI images of rectal tumors yet. As demonstrated in Fig. 3, the overview of the segmentation pipeline comprised four major stages, including preprocessing, data augmentation, model training, and post-processing, which was capable of automatic network configuration.

**Fig. 4:**
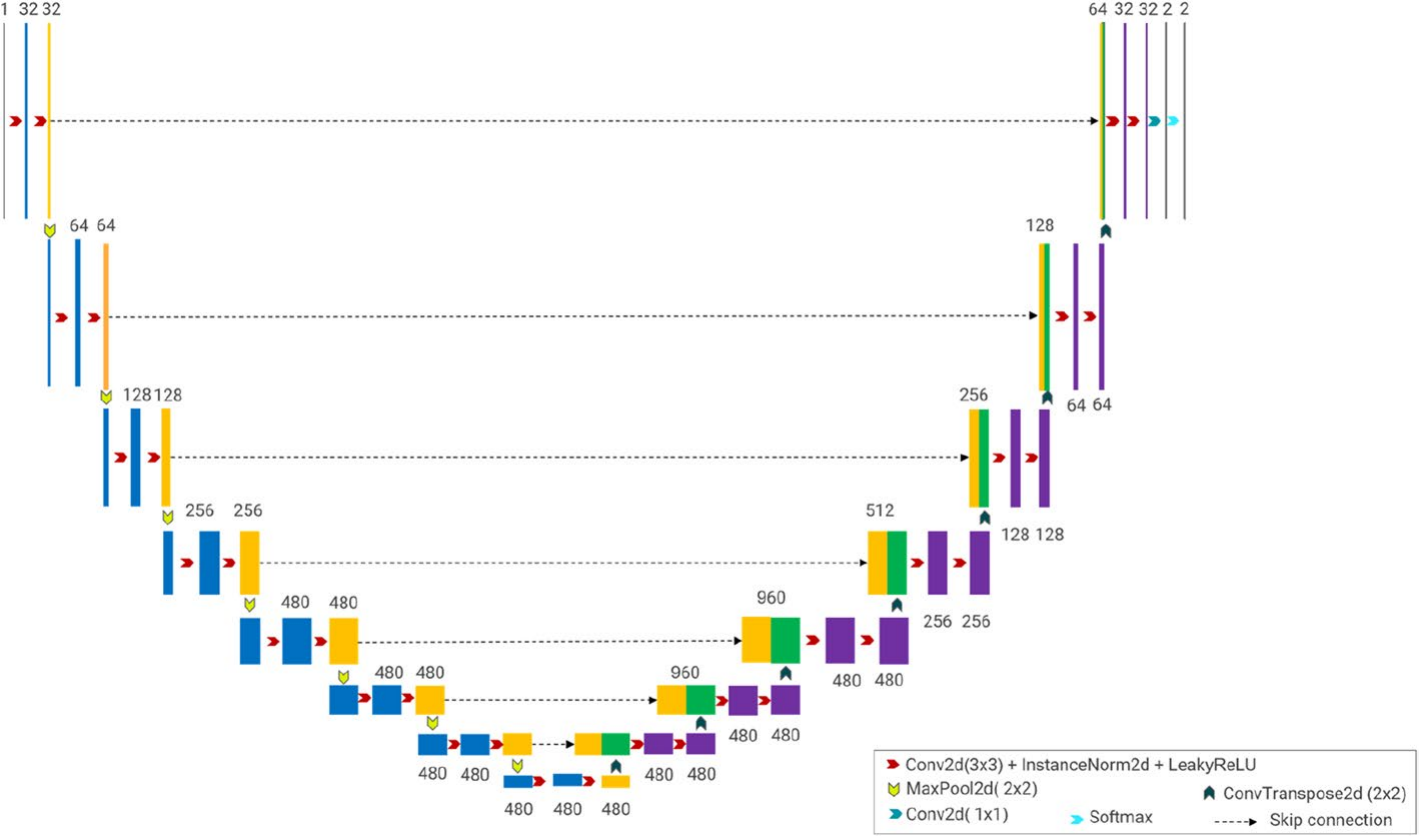
Deep convolutional network architecture

In more detail, the overall segmentation network structure was symmetrically composed of eight layers, as shown in Fig. 4, extracting and reassembling features through network structure and parameter configuration. A typical block was operated twice at every layer, which comprised a 3 × 3 convolution [stride = (1, 1), padding= (1, 1)], instance norm (eps = 1e-05, momentum = 0.1) and Leaky ReLu (negative slope = 0.01). The origin slice (1, 512, 512) passed through the convolution block, and the dimensions were converted into (32, 256, 256), (64, 128, 128), (128, 64, 64), and (256, 32, 32) from the first to the fourth layer, sequentially. Afterward, the width and height were continuously squeezed by the max pooling layer, but the number of channels no longer changed and remained at 480 in the last three layers. In the opposite direction, the feature dimensions of the decoding side changed similarly, and the feature fusion was performed with the skip layers. At the end, a 1 × 1 convolution and a softmax layer were implemented to the network, generating the predicted ROI results. Our source code is available via GitHub (https://github.com/Post-nCRT/Segmentation-of-rectal-cancer) and can be coordinated with the nnUNet code.

### Evaluation

We calculated the most commonly used metrics based on prediction results and the gold standard of doctors to quantitatively evaluate the performance of the DL model. DSC, Jaccard, Recall, Precision, and F1-score were used to measure the performance in the training stage, and DSC, HD95, and MSD were applied as the main indexes to examine the test dataset [38]. All the formulas were expressed as follows:

- ① DSC (Dice similarity coefficient): DSC is usually used to calculate the volume overlap between two sets with a value range of [0,1], where $M\cap N$ represents the intersection of the ground truth (N) and prediction (M), and | | represents the number of elements.
  1 $$DSC=\frac{2\left|M\cap N\right|}{\left|M\right|+\left|N\right|}$$
- ② Jaccard (Jaccard similarity coefficient): Given two sets $M$ and $N$, the Jaccard coefficient is defined as the ratio of the intersection of $M$ and $N$ to the union of $M$ and $N$.
  2 $$Jaccard=\frac{\left|M\cap N\right|}{\left|M\right|+\left|N\right|-\left|M\cap N\right|}$$
- ③ Recall (R): Recall is defined as the proportion of true-positive samples detected in all positive samples. Its value is equivalent to sensitivity.
  3 $$Recall=\frac{TP}{TP+FN}=\frac{\left|M\cap N\right|}{\left|N\right|}$$
- ④ Precision (P): Precision essentially measures the proportion of the true-positive samples among all samples predicted to be positive.
  4 $$Precision =\frac{TP}{TP+FP}$$
- ⑤ F1-score: The ${F}_{\beta }-score$ considers precision and recall together, and the F1-score is the harmonic mean of precision and recall, which can be expressed as Eq. 6.
  5 $${F}_{\beta }-score=\left(1+{\beta }^{2}\right)\frac{PR}{{(\beta }^{2}P+R)}$$
  6 $${F}_{1}-score=\frac{2PR}{P+R}$$
- ⑥ HD95 (95% Hausdorff distance): HD95 mainly measures the maximum distance between the ground truth (N) and prediction (M), where $hd\left(M,N\right)$ and $hd\left(N,M\right)$ are the unidirectional Hausdorff distances from set A to set B and from set B to set A, respectively. And ${K}_{95\%}$ represents the 95th percentile.
  7 $$HD95\left(M,N\right)={K}_{95\%}({\text{max}}(hd\left(M,N\right),hd\left(N,M\right)))$$
  8 $$hd\left(M,N\right)=\underset{m\in M}{{\text{max}}}\underset{n\in N}{{\text{min}}}\left|\left|m-n\right|\right|$$
  9 $$hd\left(N,M\right)=\underset{n\in N}{{\text{min}}}\underset{m\in M}{{\text{max}}}\left|\left|n-m\right|\right|$$
- ⑦ MSD (Mean surface distance): MSD mainly measures the mean distance between the two surfaces, where $d(v,S(K))$ denotes the shortest distance of an arbitrary volume $v$ to $S(K)$.
  10 $$MSD\left(M,N\right)=\frac{1}{2}\left(\frac{1}{\left|S\left(M\right)\right|}\sum_{{s}_{M}\in S\left(M\right)}d\left({s}_{M},S\left(N\right)\right)+\frac{1}{\left|S\left(N\right)\right|}\sum_{{s}_{N}\in S\left(N\right)}d\left({s}_{N},S\left(M\right)\right)\right)$$
  11 $$d\left(v,S\left(K\right)\right)=\underset{{s}_{k}\in S\left(K\right)}{{\text{min}}}\left|\left|v-{s}_{K}\right|\right|$$
- ⑧ ICC (Intraclass correlation coefficient): ICC is applied to evaluate the reliability between multiple measurements of the same object, where ${MS}_{group}$ and ${MS}_{error}$ respectively represent the mean squares of group and error, $U$ is defined as the number of measurements.
  12 $$ICC=\frac{{(MS}_{group}-{MS}_{error})/U}{{(MS}_{group}-{MS}_{error})/U+{MS}_{error}}$$

## Results

### Clinical characteristics of patients with LARC

A total of 372 patients with LARC were selected as preliminary candidates, and 284 patients (243 in the training cohort, mean age 56.37 ± 9.83 years; 41 in the test cohort, mean age 55.59 ± 11.66 years) were eventually enrolled in the study. The clinical characteristics of patients in the training and test cohorts, including number of MRI slices, age, sex, and TRG levels, are summarized in Table 1.

**Table 1:** Clinical records of patients with LARC

Characteristics	Training dataset	Test dataset
Number of patients	243	41
Number of slices	3061	408
Sex (female/male)	101/142	15/ 26
Mean age (years)	56.37 ± 9.83	55.59 ± 11.66
TRG (TRG1/TRG2/TRG3)	107/131/5	18/19/4

### Model training and evaluation

The network architecture was developed and trained on a workstation with two GeForce RTX 2080 GPUs (Python 3.7, PyTorch 1.7.1, Linux system, ubuntu 16.04 server). The total training epochs were set to 500, and the initial learning rate was set to 1e-3, optimized by stochastic gradient descent. The five common evaluation metrics - DSC, Jaccard, Precision, Recall, and F1-score of fivefold cross-validation are summarized in Table 2. By calculating with the gold standard of doctors in each fold (20% used for validation), the developed model achieved a mean DSC, Jaccard, Precision, Recall, and F1-score of 0.881 (95% CI: 0.879–0.884), 0.798 (95% CI: 0.795–0.802), 0.880 (95% CI: 0.876–0.884), 0.899 (95% CI: 0.898–0.900), and 0.881 (95% CI: 0.879–0.884), respectively.

**Table 2:** Evaluation metrics (DSC, Jaccard, Precision, Recall, and F1-score) with fivefold cross-validation

Fold	DSC	Jaccard	Precision	Recall	F1-score
0	0.878 (0.871–0.886)	0.794 (0.784–0.805)	0.875 (0.865–0.885)	0.898 (0.891–0.906)	0.878 (0.871–0.886)
1	0.885 (0.878–0.893)	0.804 (0.795–0.814)	0.886 (0.876–0.895)	0.900 (0.894–0.906)	0.885 (0.878–0.893)
2	0.882 (0.875–0.889)	0.798 (0.789–0.808)	0.882 (0.873–0.891)	0.897 (0.890–0.904)	0.882 (0.875–0.889)
3	0.879 (0.871–0.887)	0.796 (0.786–0.806)	0.878 (0.868–0.887)	0.899 (0.891–0.906)	0.879 (0.871–0.887)
4	0.881 (0.874–0.889)	0.799 (0.789–0.809)	0.880 (0.870–0.890)	0.899 (0.892–0.906)	0.881 (0.874–0.889)
Mean	0.881 (0.879–0.884)	0.798 (0.795–0.802)	0.880 (0.876–0.884)	0.899 (0.898–0.900)	0.881 (0.879–0.884)

**Fig. 5:**
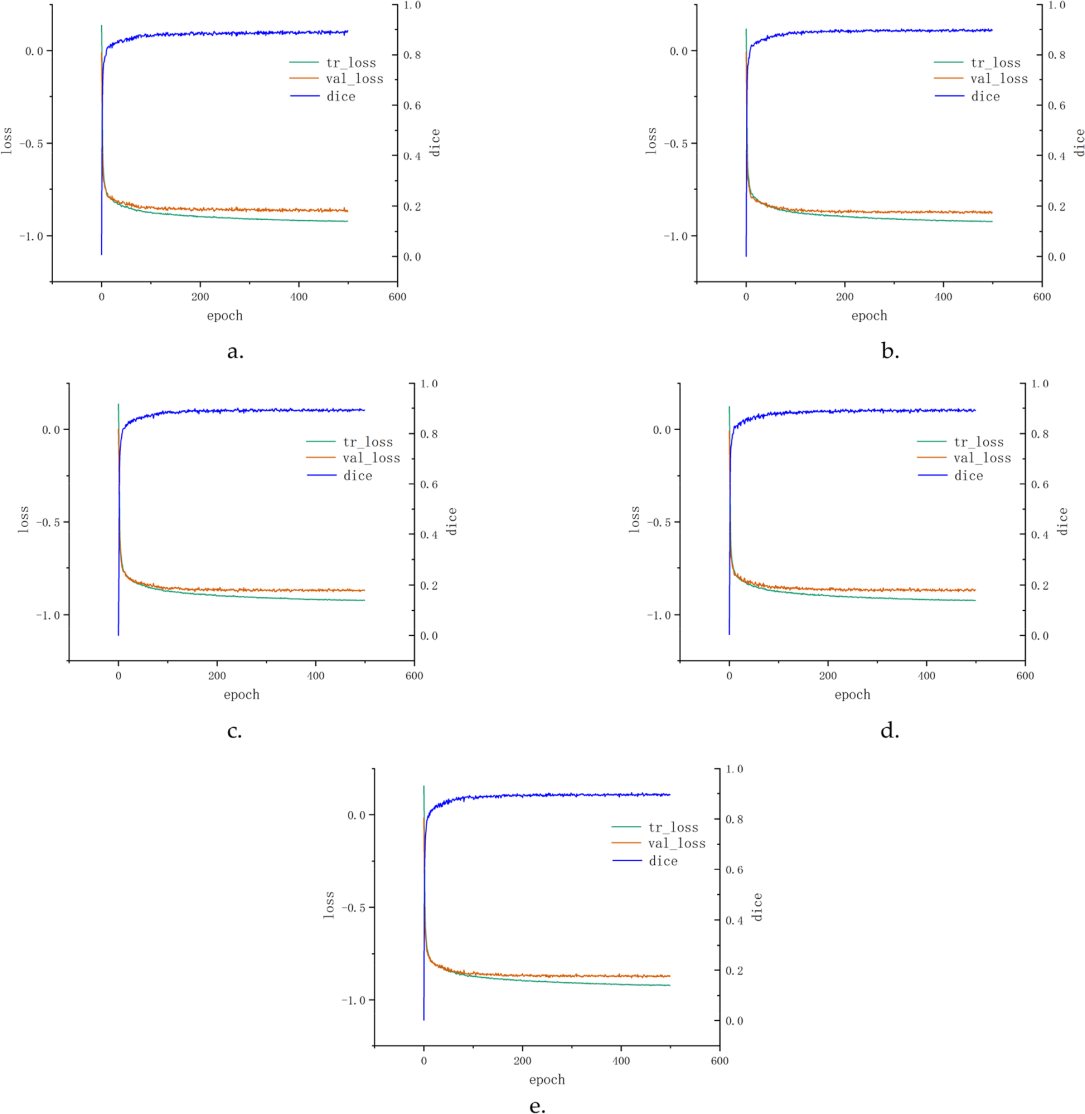
Learning curves of fivefold cross-validation: (1) **a**-**e** Loss graphs and evaluation metrics (DSC) from first fold to fifth fold. (2) Left axis: changes in losses on training and validation dataset from 0 to 499 epochs. (3) Right axis: DSC values on the validation dataset from 0 to 499 epochs

The learning curves of the first fold to fifth fold are depicted in Fig. 5. The changes in training and validation losses were measured using the scale of the left axis, and DSC values on the validation dataset were visualized using the right axis. From 0 to 200 epochs, the DSC values smoothly increased and then gradually stabilized at 0.88 after 200 epochs.

### Model performance in the test dataset

The trained DL model was examined on 408 slices with LARC. And the results are demonstrated in Table 3. The mean DSC, mean HD95, and mean MSD were 0.700(95% CI: 0.680–0.720), 17.73 mm (95% CI: 16.08–19.39), and 3.11 mm (95% CI: 2.67–3.56), respectively. Considering for HD95, 122 slices (30%) were less than 5 mm, and 225 slices (55%) were less than 15 mm. Simultaneously, the MSD values of 334 slices (82%) were less than 5 mm, of which 224 slices (55%) were less than 2 mm.

**Table 3:** Evaluation metrics (DSC, HD95, and MSD) for the test dataset on different TRGs

TRG grade	Slices	Statistics	DSC	HD95 (mm)	MSD (mm)
TOTAL	408	Mean	0.700 (0.680–0.720)	17.73 (16.08–19.39)	3.11 (2.67–3.56)
		Median	0.750	12.98	1.62
		Maximum	0.960	85.38	52.11
		Minimum	0.000	0.00	0.07
TRG1	175	Mean	0.670 (0.640–0.700)	19.73 (17.33–22.13)	2.32 (1.83–2.80)
		Median	0.690	16.12	2.20
		Maximum	0.930	75.80	19.29
		Minimum	0.140	1.00	0.13
TRG2	199	Mean	0.720 (0.690–0.750)	15.41 (13.03–17.79)	3.02 (2.23–3.81)
		Median	0.790	8.98	1.11
		Maximum	0.960	85.38	52.11
		Minimum	0.00	0.00	0.07
TRG3	34	Mean	0.690 (0.620–0.760)	21.08 (14.67–27.50)	3.45 (2.18–4.72)
		Median	0.760	12.35	1.43
		Maximum	0.930	55.07	13.82
		Minimum	0.270	1.00	0.12

**Fig. 6:**
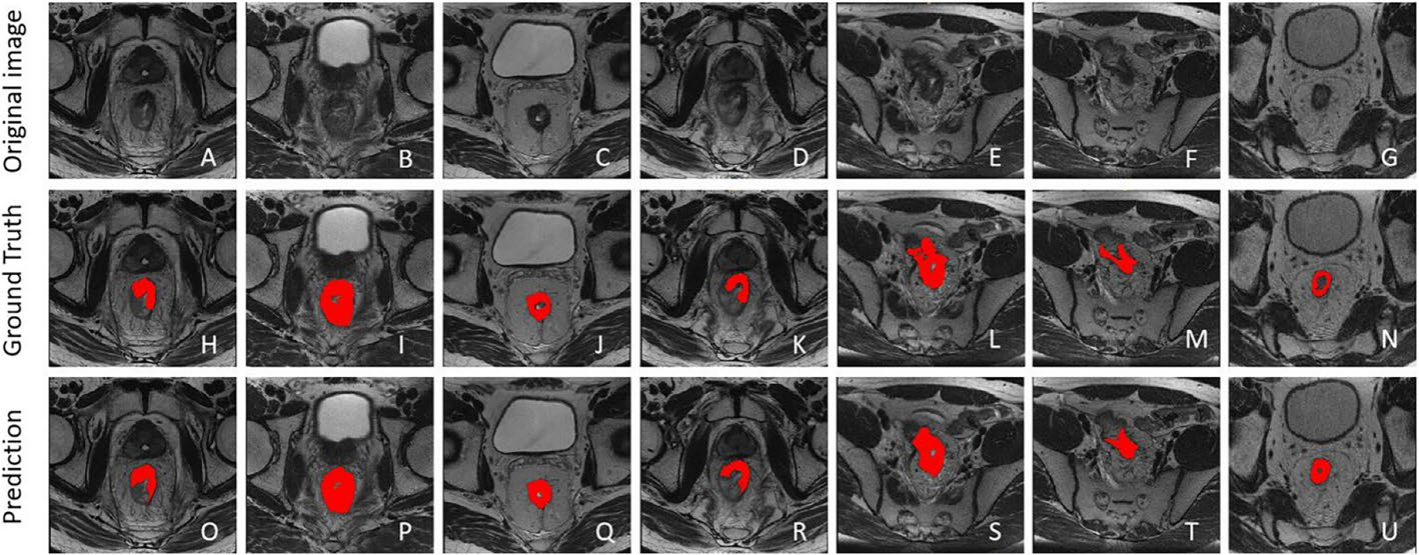
Examples of comparison between segmentation results of the DL model and the annotations from radiologists: **a**–**g** original images; **h**–**n** ground truth; and **o**–**u** prediction results. The red areas represent ROIs

The examples of segmentation results were compared with the original images and segmention output, as shown in Fig. 6, in which the red areas represent ROIs. (a–g) are original images, (h–n) are ground truth annotations by radiologists, and (o–u) are prediction results by the deep convolutional network.

**Fig. 7:**
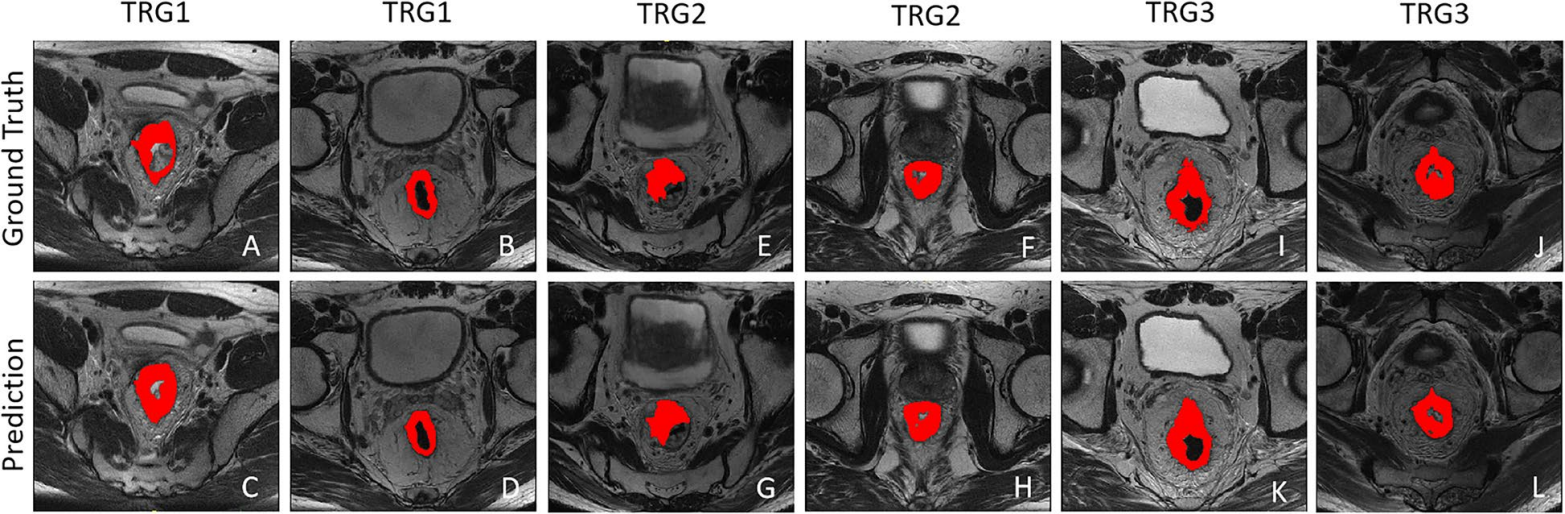
Segmentation examples of tumor lesions with different TRGs. **a**–**d** TRG1; **e**–**h** TRG2; and **i**–**l** TRG3. The red areas represent ROIs

To evaluate the segmentation performance of tumors with various changes on post-nCRT, the quantitative evaluation results for patients on different TRGs were also independently calculated, as shown in Table 3. The average values of DSC, HD95, and MSD were (0.670 [95% CI: 0.640–0.700], 19.73 mm [95% CI: 17.33–22.13], and 2.32 mm [95% CI: 1.83–2.80]), (0.720 [95% CI: 0.690–0.750], 15.41 mm [95% CI: 13.03–17.79], and 3.02 mm [95% CI: 2.23–3.81]), and (0.690 [95% CI: 0.620–0.760], 21.08 mm [95% CI: 14.67–27.50], and 3.45 mm [95% CI: 2.18, 4.72]), respectively. When further assessing the test results for each TRG type, the model exhibited consistent DSC values within the range of 70% ± 3%, and demonstrated the stability of the pixel-level overlaps of inference results and ground truth on different TRG levels. And it was evident that the values of HD95 and MSD basically increased from TRG1 to TRG3, disregarding the potential decrease in HD95 caused by a higher training and testing slices of TRG2. The rise of the two metrics indicated that the segmentation of tumor surface boundaries became more challenging as the degree of tumor regression increased after nCRT, which was also aligned with the practical experience on manual delineation. Fig. 7(a–l) visually shows the segmentation examples of tumor lesions, each TRG level is illustrated with two cases, with the comparison of both the prediction results of DL model and the ground truth from radiologists.

Furthermore, statistical analyses were conducted to provide more adequate comparability of DSC. The intraclass correlation coefficient (ICC) of the representative radiomics feature, the maximum diameter, was computed by pyradiomics 3.0.1 and SPSS Statistics 27.0 (IBM official version). In Table 4, the ICC between expert readers, the ICC between expert readers and deep learning model, and the ICC mentioned by previous literature [24], are summarized together to provide quantitative explanations for the difficulty of rectal tumor segmentation on post-nCRT. The ICC of the same lesion areas delineated by radiologists and predicted by deep learning model was 0.669 (95%CI: 0.612, 0.719), comparing the interreader agreement on T2 images between the two human radiologists with the value of 0.739 (95% CI: 0.515, 0.865).

**Table 4:** ICCs for assessment of task difficulty: the ICC between expert readers, the ICC between expert readers and deep learning model, and the ICC mentioned by previous literature [24]

Two objects	Post-nCRT ICC
Two human radiologists (T2WI)	0.739 (95% CI: 0.515, 0.865)
DL model and human radiologists (T2WI)	0.669 (95%CI: 0.612, 0.719)
Two human radiologists [24] (DWI)	0.750 (95%CI: 0.630, 0.830)
Automated segmentation using the software [24] (DWI)	0.530–0.660
Semiautomated segmentation using the software [24] (DWI)	0.610–0.750

## Discussion

The automatic segmentation of rectal tumors on post-nCRT MRI makes a positive contribution to the evaluation of the nCRT effect, which is also the footstone of the subsequent processes, including tumor measurement, radiomics analysis, surgical plan decision, and so forth. When only post-nCRT MRI images are available, it is particularly critical to ensure the reliability and accuracy of segmentation results. A high probability exists that confounding factors would be introduced if the images of patients with pCR (theoretically accounting for 20%) were directly sent to the segmentation model for training [11]. The clinicians could neither delineate the ROI nor equate it with a completely tumor-free region. Thus, the patients with pCR were first excluded, and only patients without pCR (243 with 3061 slices) were used to construct the segmentation model.

In this study, we mainly focused on post-nCRT MRI alone and developed a fully automatic pipeline using the state-of-the-art framework, nnUNet. The experimental results of the DL model showed a high segmentation accuracy in 5-fold cross-validation, with a mean DSC, Jaccard, Precision, Recall, and F1-score of 0.881 (95% CI: 0.879–0.884), 0.798 (95% CI: 0.795–0.802), 0.880 (95% CI: 0.876–0.884), 0.899 (95% CI: 0.898–0.900), and 0.881 (95% CI: 0.879–0.884), respectively. The mean DSC value was maintained at 70% on the test dataset (41 patients, 408 slices).

The tumors generally shrink after chemoradiotherapy and lesion areas are usually accompanied by varying degrees of fibrosis, and hence the segmentation on post-nCRT is more challenging than the segmentation on baseline MRI. TRG is mainly graded according to the residual tumor components and the proportion of fibrosis. Thus, the therapeutic effects of chemotherapy and targeted drugs on tumors can be quantitatively analyzed. We calculated the metrics of the DL model at different TRG levels (TRG1, TRG2, and TRG3) to further evaluate the generalization performance. The mean DSC, mean HD95, and mean MSD were 0.670 (95% CI: 0.640–0.700), 19.73 mm (95% CI: 17.33–22.13), and 5.98 mm (95% CI: 5.00– 6.96); 0.720 (95% CI: 0.690–0.750), 15.41 mm (95% CI: 13.03, 17.79), and 4.74 mm (95% CI: 3.54–5.94); and 0.690 (95% CI: 0.620–0.760), 21.08 mm (95% CI: 14.67, 27.50), and 6.47 mm (95% CI: 3.89, 9.05), respectively.

Previous studies included cases only related to post-nCRT MRI images involving segmentation of the rectal wall or suspicious areas on post-nCRT [22–24]. Still, they were not directly related to the segmentation of tumor areas. Thomas et al. [22] trained a fully convoluted network for the segmentation of the rectal wall on post-chemoradiation T2-weighted MRI, and the median DSC reached 0.680. Pang et al. [23] employed both U-Net and 4-channel U-Net on “suspicious region” segmentation for follow-up radiomics analysis, achieving DSC values of 0.656 (95% CI: 0.630–0.683) and 0.660 (95% CI: 0.628–0.691), respectively. Meanwhile, compared with the manual method, the trained DL model showed better performance than either automated or semiautomated segmentation using the software with DSC of 0.420 ± 0.230 (ICC: 0.530~0.660) and 0.410 ± 0.220 (ICC: 0.610~0.750) [24], respectively.

Although relatively stable results were obtained in this study, it still has some limitations for future improvement and optimization. From the perspective of the dataset, we could recruit patients on each TRG grade as much as possible to ensure a more balanced sample distribution from different TRGs. Additionally, the DL model trained on the retrospective dataset could be further validated on a prospective multicenter dataset. In light of the diminishing likelihood of obtaining validation through anatomopathological reports due to the increasing use of the watch-and-wait protocol and the option of local excision [39, 40], next endeavors will be laid on exploring weakly supervised or unsupervised artificial intelligence approaches in the scenario of few pathological labels [41–43]. And tissue specimens from appropriate patients with local excision can also be obtained for pathologic study, with less differences from the patients that undergo TME surgery.

Deducing the growth sorely from model promotion and imaging technology, it is considered that introducing multi-stage segmentation steps or attention mechanisms may increase the segmentation accuracy. Furthermore, the developing application of the suitable integration of 2D and 3D models [44, 45] in diverse clinical scenes will be the desired research direction. As post-nCRT imaging techniques for rectal cancer continue to advance, investigating the automated segmentation performance through multimodal imaging technologies such as PET/CT or PET/MRI also represents a promising avenue [16, 46].

## Conclusions

In this study, we developed an automatic segmentation pipeline for LARC exclusively based on post-nCRT T2-weighted MRI. It was the first attempt to evaluate and validate the application potential of nnUNet framework for rectal cancer on post-nCRT MRI imaging, differing from CT slices in previous studies. The experimental results indicated a relatively high accuracy (DSC, HD95, and MSD). Moreover, the robustness of the network was also verified by analyzing the segmented tumor lesions on diverse TRGs. The model is expected to be not only an auxiliary tool for manual labeling but also a potential practical tool for subsequent tumor measurement, radiomics analysis, treatment planning, and operative strategy with further multicentre external validation. Future studies will focus on exploring effective methods to combine 2D models with 3D models and further apply them to clinical populations.

## Data Availability

The datasets are available from the first author (SX) and corresponding author on reasonable request.

## Abbreviations

LARC: Locally advanced rectal cancer
nCRT: Neoadjuvant chemoradiotherapy
TME: Total mesorectal excision
pCR: Pathologic complete response
MRI: Magnetic resonance imaging
T2WI: T2-weighted imaging
DWI: Diffusion-weighted imaging
CT: Computed tomography
TRG: Tumor regression grade
DL: Deep learning
DSC: Dice similarity coefficient
HD95: 95% Hausdorff distance
MSD: Mean surface distance
CI: Confidence interval
ROIs: Regions of interest
ICC: Intraclass correlation coefficient
